# The impact of immunosuppressive therapy on secondary infections and antimicrobial use in COVID-19 inpatients: a retrospective cohort study

**DOI:** 10.1186/s12879-023-08697-9

**Published:** 2023-11-17

**Authors:** Peter Crook, Clare Logan, Andrea Mazzella, Rachel M. Wake, Martina Cusinato, Ting Yau, Yee-Ean Ong, Timothy Planche, Marina Basarab, Tihana Bicanic

**Affiliations:** 1 St George’s University Hospitals NHS Foundation Trust, Blackshaw Road, London, SW17 0QT UK; 2 Institute of Infection & Immunity, St George’s, University London, Cranmer Terrace, London, SW17 0RE UK; 3 Institute of Medical and Biomedical Education, St. George’s, University of London, Cranmer Terrace, London, SW17 0RE UK

**Keywords:** COVID-19, Coinfection, Immunosuppressive agents, Steroids, Antimicrobial stewardship

## Abstract

**Background:**

Immunosuppressive therapies have become a cornerstone of the management of severe COVID-19. The impact of these therapies on secondary infections and antimicrobial prescribing remains unclear. We sought to assess antimicrobial use and the incidence of bacterial and fungal infections in patients with severe COVID-19, and to explore their associations with receipt of immunosuppressive therapies.

**Methods:**

Our retrospective cohort study included 715 hospitalised, adult patients with severe COVID-19 admitted to St George’s Hospital, London, UK, during the first UK pandemic wave (1^st^ March–10^th^ June 2020). Co-infections (occurring within 48 h of admission) and secondary infections (≥ 48 h) were defined as a positive microbiological culture with supporting clinical, radiological or laboratory data to suggest true infection. Cox regression models with time-dependent covariates were used to explore the association between immunosuppressant use and secondary infection.

**Results:**

Microbiologically confirmed co-infection occurred in 4.2% (*n* = 30) and secondary infection in 9.3% (*n* = 66) of the cohort (*n* = 715) and were associated with in-hospital mortality (48% vs 35%, OR 1.8, 95%CI 1.1–2.7, *p* = 0.01). Respiratory (*n* = 41, 39%) and bloodstream infections (*n* = 38, 36%) predominated, with primarily Gram-negative pathogens. 606 (84.7%) patients received an antimicrobial, amounting to 742 days of therapy per 1000 patient-days (DOTs). In multivariable models, receipt of high-dose steroids (≥ 30 mg prednisolone or equivalent) or tocilizumab was significantly associated with increased antimicrobial consumption (+ 5.5 DOTs, 95%CI 3.4–7.7 days) but not secondary infection (HR 0.56, 95%CI 0.26–1.18).

**Conclusions:**

Bacterial and fungal infections in severe COVID-19 were uncommon. Receipt of steroids or tocilizumab was independently associated with antimicrobial consumption despite its lack of association with secondary infection. These findings should galvanise efforts to promote antimicrobial stewardship in patients with COVID-19.

**Supplementary Information:**

The online version contains supplementary material available at 10.1186/s12879-023-08697-9.

## Background

Bacterial and fungal infections are a recognised complication of respiratory viral illnesses, occurring in ~ 23% [1] and ~ 10% [2] of severe influenza patients respectively, and are associated with critical illness and mortality. Bacterial and fungal infections in COVID-19, however, appear to be less frequent (8.8%) [3] and are largely hospital-acquired [3–6]. Despite this, antimicrobial prescribing early in the pandemic was reported to be ~ 75–85% [4, 7], although detailed class-specific antimicrobial consumption data are scarce.

Few data exist regarding the impact of immunomodulatory therapies on the incidence of secondary infection and antimicrobial prescribing practice. Immunomodulatory drugs including steroids and interleukin 6 (IL-6) inhibitors are now recommended to manage dysfunctional hyperinflammation in severe COVID-19 [8]. The cytokine signalling pathways blocked by IL-6 inhibition, coupled with the inhibitory effect of steroids on neutrophil and macrophage function, may impair local and systemic immune responses to infection [9, 10], potentially increasing the risk of secondary infection. In turn, this perceived increased infection risk may also affect antimicrobial prescribing practice for patients with COVID-19.

The aims of our study were to identify the incidence of co- and secondary bacterial and fungal infections in hospitalized patients with severe COVID-19 during the first wave of the UK pandemic; to assess antimicrobial consumption; and to identify any association between exposure to immunosuppressive therapies and risk of COVID-related infections and antimicrobial use.

## Methods

### Study design, cohort selection and setting

This retrospective observational cohort study included adult (≥ 18 years) patients admitted to a ward or intensive care unit (ICU) at St George’s University Hospital, a 1000-bed teaching hospital in London, UK, with confirmed, severe COVID-19 infection between 1^st^ March and 10^th^ June 2020.

Severe COVID-19 was defined as a positive SARS-CoV-2 PCR and either a new oxygen requirement or oxygen saturation < 94%. We excluded inter-hospital transfers due to risk of missing data. The follow-up period commenced 7 days before the first positive SARS-CoV-2 swab (or date of hospital admission, if later) and ended either on hospital discharge or 60 days following the diagnostic swab, whichever was earlier.

### Data collection

Potential participants were identified retrospectively using hospital records of all admissions to dedicated COVID-19 areas. Data were extracted from electronic patient records to identify demographics, comorbidities, observations, ICU admission, receipt of organ support and outcome (death or discharge). Microbiology data were extracted manually from the laboratory information management system for culture, PCR and antigen test results from any site. Receipt of antimicrobials and immunosuppressive therapy (Table S1) were extracted from electronic prescribing records.

### COVID-related infections

We defined COVID-related infection as a microbiologically confirmed infection occurring during the follow-up period. Infections were classified as co-infections if they occurred prior to or within 48 h of the first positive SARS-CoV-2 PCR swab, and secondary infections if they occurred after 48 h.

We excluded non-pathogenic organisms from non-sterile sites (see S2 for list). For all remaining positive cultures, a detailed manual review of clinical, radiological and laboratory data from electronic patient records was conducted by one of five infectious diseases and microbiology physicians (PC, CL, RMW, MB, TB; see S3 for data collection methods). A culture was deemed significant if the organism identified was a potential pathogen and there was accompanying evidence of true infection. In the absence of such evidence, positive cultures were deemed commensals or contaminants. Equivocal cases were discussed by the whole panel of infection specialists and a consensus reached. For bloodstream infections, the source was determined based on clinical and radiological findings, along with corresponding cultures from other sites.

### Antimicrobial use

Antimicrobial Days of Therapy (DOT) were calculated for each antimicrobial agent and each patient. Antimicrobial consumption was expressed using DOT per 1000 patient-days of follow-up; this metric accounts for time-at-risk and mitigates any survival bias between subgroups. We used the AWaRe England Antimicrobial classification, which categorises antimicrobials into three groups: i) ‘Access’, comprising common, empirical antimicrobial choices; ii) ‘Watch’, for antimicrobials whose use requires monitoring due to higher toxicity or resistance concerns; and iii) ‘Reserve’, for antimicrobials that should be reserved for complex or multidrug-resistant infections only [11]. Antifungal agents are not included in the AWaRe classification and are presented separately.

The local prescribing policy during the first wave of the pandemic was doxycycline for all COVID-19 patients requiring oxygen, with additional intravenous amoxicillin for those with a respiratory rate > 20 breaths per minute or oxygen requirement of ≥ 40%.

### Exposure to immunosuppressive therapy

Exposure to high-dose immunosuppressive therapy was defined as receipt of at least two doses of ≥ 30 mg prednisolone (or equivalent) or receipt of tocilizumab within the follow-up period (S1).

### Statistical analysis

Incidence of infection was calculated as a rate (number of events over patient-days of follow-up) with Poisson 95% confidence intervals (CI) using an exact method.

Univariable analyses for COVID-related infections, days of follow-up and mortality were performed using Wilcoxon rank sum tests for quantitative variables and chi-square or Fisher’s exact tests for categorical variables. The 95% confidence interval (CI) around the odds ratio of mortality and infection were calculated using Wald’s method of normal approximation.

Cox regression models with robust variance were used to explore the association between infection (outcome) and prior immunosuppression (exposure). Exposure to immunosuppression was specified as a time-dependant covariate, as infections could occur throughout the hospital admission, either before or after exposure to immunosuppressive therapy. Robust variance was used to account for repeated outcomes, as a small proportion of participants developed two infections. A univariable model was followed by a multivariable model adjusting for ICU admission and age.

The association between antimicrobial DOT (outcome) and immunosuppression (exposure) was assessed using linear regression (adjusting for ICU admission, co- or secondary infection, and length of follow-up in the multivariable model). Models restricted to each antimicrobial category of the AWaRe England Antimicrobial classifications were fitted.

We selected variables to include in the multivariable regression models based on an *a priori* decision, supported by directed acyclic graphs. There were no missing data in the variables used in the regression models.

Statistical analyses were performed using R version 4.1.2 (R Foundation for Statistical Computing) using libraries: tidyverse, gtsummary, epitools, and survival.

### Ethics

The study was granted ethical approval by the Health Research Authority (20/SC/0220).

## Results

### Overview of the cohort

**Fig. 1:**
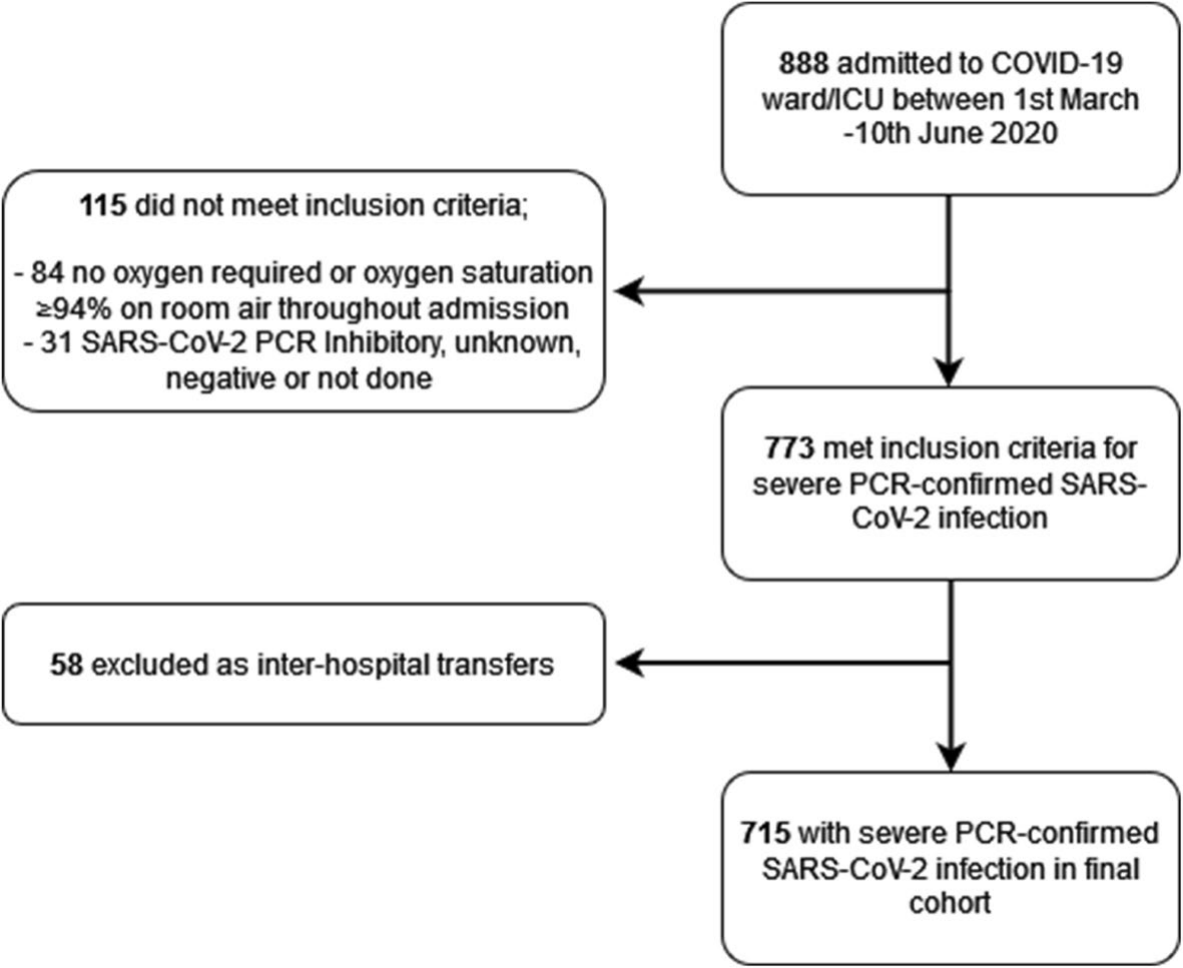
Flow chart of participant inclusion in cohort

From 1^st^ March–10^th^ June 2020, 888 adult patients were admitted to a designated COVID-19 ward or ICU; 773 met the inclusion criteria of PCR-confirmed, severe SARS-CoV-2 infection; 58 inter-hospital transfers were excluded; the final cohort comprised of 715 patients with a total follow-up period of 10,119 patient-days (Fig. 1). Demographic and clinical characteristics of the cohort are summarised in Table 1. Median age was 74 years, 58% were male, with the most common co-morbidities of diabetes (31%) and chronic lung disease (17%). ICU admission occurred in 19%, and 15% of the cohort were mechanically ventilated, with an overall in-hospital mortality of 37%.

**Table 1:** Clinical characteristics of the cohort

**Characteristic**		**Overall, *N* = 715**^**1**^	**Co- or secondary infections**		***p*-value**
			**None, *N* = 620**^**1**^	**One or more, *N* = 95**^**1**^	
**Demographics**	Age [years]	74 (59, 83)	75 (61, 84)	66 (55, 78)	< 0.001^2^
	Male gender	414 (58%)	358 (58%)	56 (59%)	0.8^3^
**Comorbidities**	Charlson comorbidity index [score]	4 (2, 6)	4 (3, 6)	3 (2, 5)	0.002^2^
	Ischaemic heart disease	113 (16%)	102 (16%)	11 (12%)	0.2^3^
	Chronic lung disease	121 (17%)	108 (18%)	13 (14%)	0.4^3^
	Diabetes mellitus	221 (31%)	190 (31%)	31 (33%)	0.7^3^
	Renal replacement therapy	31 (4%)	25 (4%)	6 (6%)	0.3^4^
	Immunocompromised^5^	30 (4%)	25 (4%)	5 (5%)	0.6^4^
	Haematological malignancy	17 (2%)	15 (2%)	2 (2%)	> 0.9^4^
**Organ support**	ICU admission	138 (19%)	82 (13%)	56 (59%)	< 0.001^3^
	Vasopressor or inotropic support	97 (14%)	46 (7%)	51 (54%)	< 0.001^3^
	Non-invasive ventilation	12 (2%)	9 (1%)	3 (3%)	0.2^4^
	Invasive ventilation	109 (15%)	55 (9%)	54 (57%)	< 0.001^3^
	Renal replacement therapy in ICU	38 (5%)	16 (3%)	22 (23%)	< 0.001^3^
**Immunosuppression during study**	Received high-dose corticosteroids	54 (8%)	44 (7%)	10 (11%)	0.2^3^
	Received tocilizumab	17 (2%)	14 (2%)	3 (3%)	0.5^4^
	Received high-dose corticosteroids or tocilizumab	67 (9%)	56 (9%)	11 (12%)	0.4^3^
**Admission outcomes**	Days of follow-up	11 (5, 18)	10 (5, 17)	18 (11, 37)	< 0.001^2^
	In-hospital mortality	261 (37%)	215 (35%)	46 (48%)	0.01^3^

^1^Median (IQR) for continuous variables; n (column % of non-missing data) for categorical variables

^2^Wilcoxon rank sum test

^3^Pearson’s Chi-squared test

^4^Fisher’s exact test

^5^Defined as exposure to chemo- or radiotherapy, prolonged high-dose steroid in previous 6 months, AIDS or congenital immunodeficiency

### COVID-related infections

During the study period, 13.3% (*n* = 95) of the cohort developed a total of 105 microbiologically confirmed infections (S4); 4.2% of the cohort had co-infection (*n* = 30) and 9.3% secondary infection (*n* = 66), with 10 participants (1.4%) having > 1 infection. The overall incidence was 10.4 infections per 1000 patient-days (95% CI: 8.5–12.6). Patients with a co- or secondary infection had 77% higher odds of in-hospital mortality (48% vs 35%, OR 1.8, 95% CI 1.1–2.7, *p* = 0.01).

Microbiological sampling was performed in 82% of the cohort (blood culture 71%, urine culture 40%, respiratory sample 16%); sampling was more common in those who were admitted to ICU (99% vs. 78% for any sample; 59% vs. 6% for respiratory samples).

**Fig. 2:**
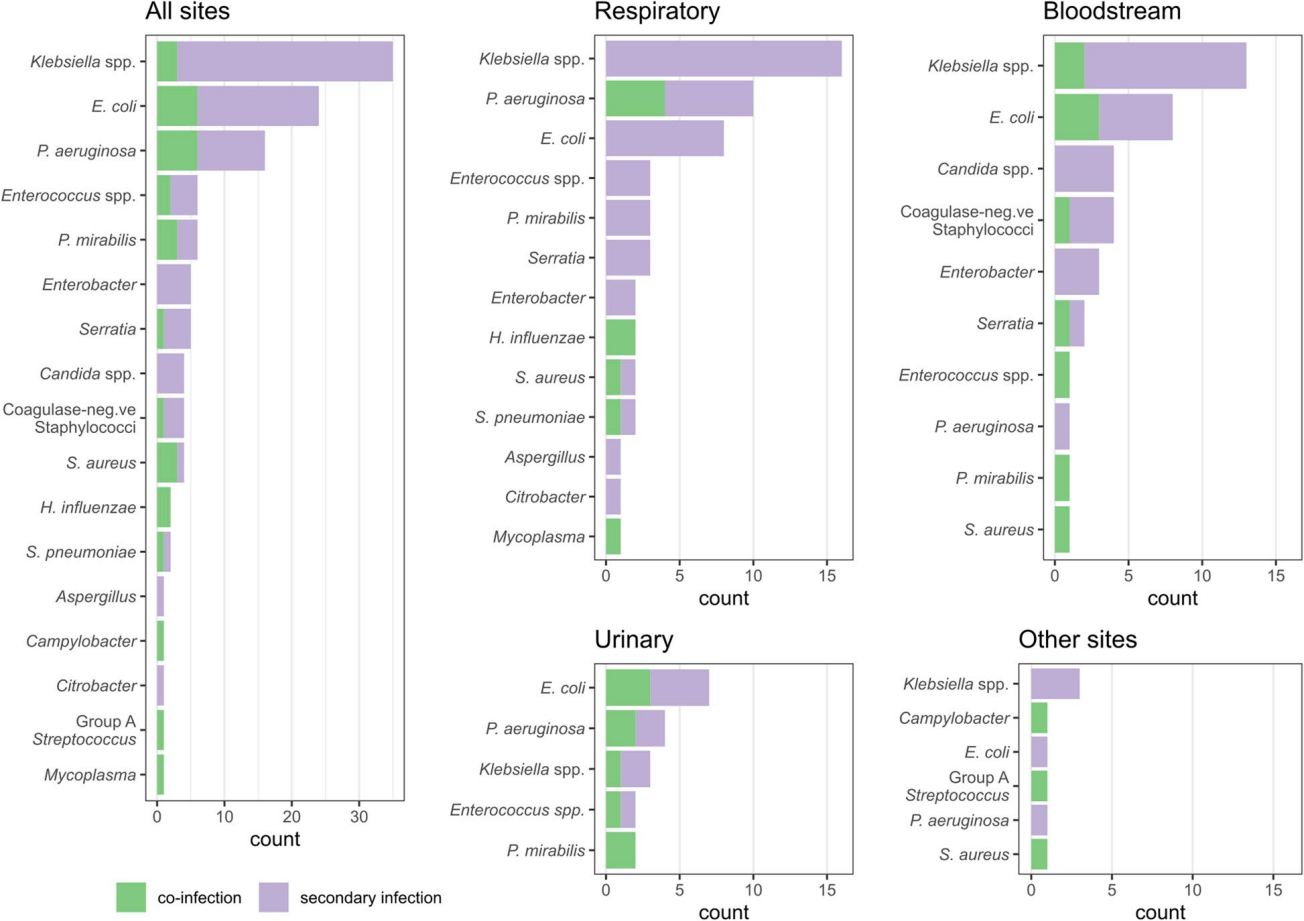
Bar chart illustrating the pathogen profile stratified by co-infection and secondary infection and by site

The most frequently identified co- and secondary infections were of the respiratory tract (*n* = 41 [39%]; incidence 4.1 per 1000 patient-days), followed by bloodstream infection (*n* = 38 [36%]; incidence 3.8 per 1000 patient-days) and urinary tract infections (*n* = 18 [17%]; incidence 1.8 per 1000 patient-days), with just 8 infections (8%) occurring at other sites (3 skin/soft tissue; 4 line infections without bacteraemia; 1 intra-abdominal). The sources of bloodstream infections were: line (*n* = 7); urinary (*n* = 5); respiratory (*n* = 4); soft tissue (*n* = 2); intra-abdominal (*n* = 1); and unknown (*n* = 19). Twelve infections (11%) involved ≥ 2 pathogens, giving a total of 118 pathogens isolated from 105 discrete infection episodes (Fig. 2).

Overall, Gram-negative secondary infections predominated. For respiratory co-infections (*n* = 9), the most common pathogens were *Pseudomonas aeruginosa* (4 [44%]) and *Haemophilus influenzae* (2 [22%]); for respiratory secondary infections (*n* = 45) the most common were: *Klebsiella* species (16 [36%]), *Escherichia coli* (8 [18%]) and *Pseudomonas aeruginosa* (6 [13%]). The most common bloodstream pathogens were: *Klebsiella* species (13 [34%]), *E. coli* (8 [21%]), *Candida* species (4 [11%]), and *Coagulase-negative staphylococci* (4 [11%]).

### Antimicrobial use

**Fig. 3:**
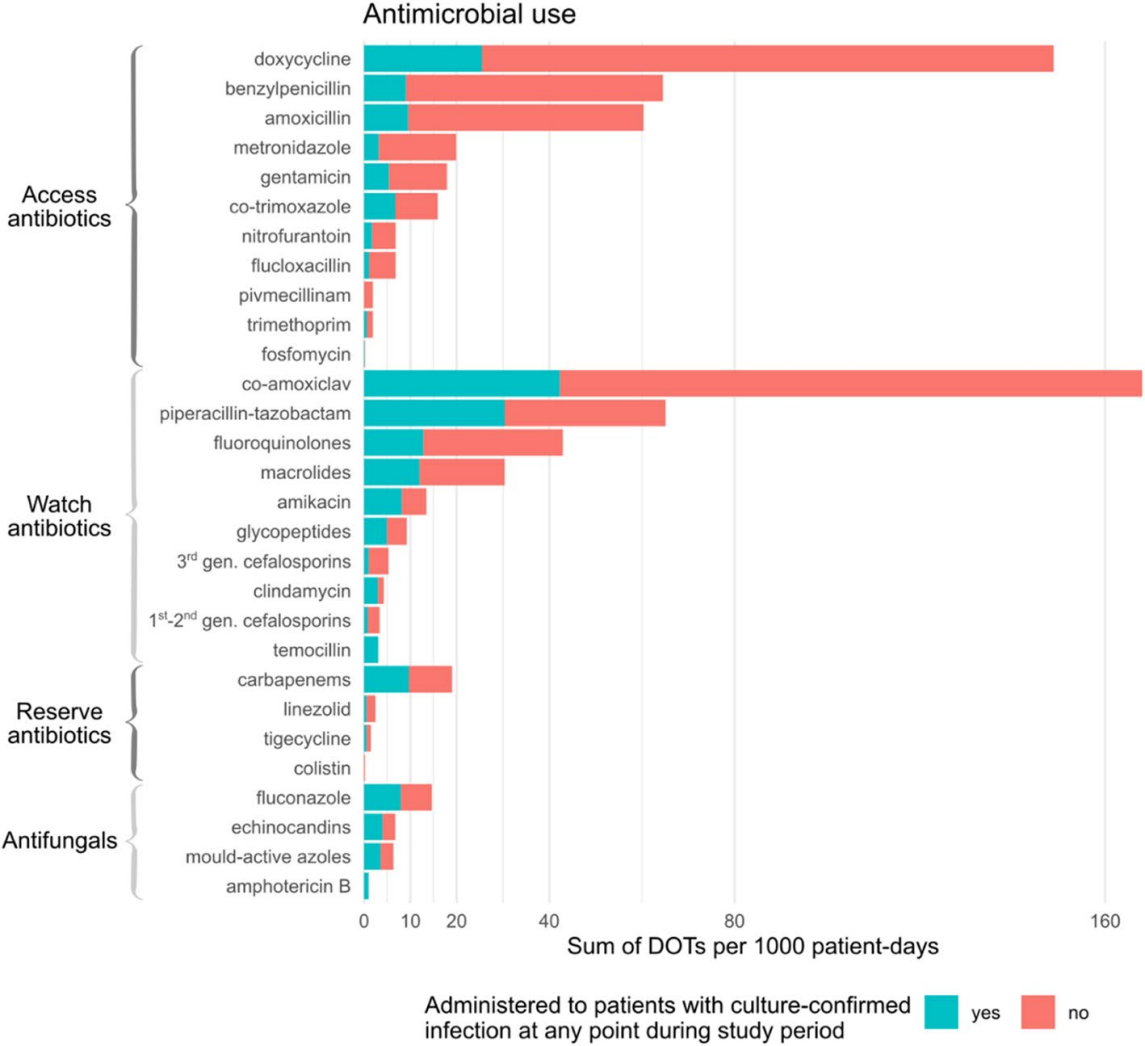
Antimicrobial use in Days of Therapy (DOT) per 1000 patient-days, stratified by antimicrobial class and presence or absence of a microbiologically confirmed infection

Antimicrobial therapy was received by 84.7% (*n* = 606) of the cohort, accounting for 742 DOT/1000 patient-days (Table 2). Figure 3 illustrates antimicrobial consumption according to antimicrobial class and presence or absence of a confirmed co- or secondary infection.

**Table 2:** Antimicrobial use stratified by receipt of immunosuppression

**Characteristic**	**Overall, ***N* = 715^1^	**High-dose immunosuppression**		***p*-value**
		**No, ***N* = 648^1^	**Yes, ***N* = 67^1^	
Received any antimicrobial	606 (85%)	542 (84%)	64 (96%)	0.01^2^
Number of antimicrobial courses	2 (1, 3)	2 (1, 3)	3 (2, 5)	< 0.001^3^
Antimicrobial Days of Treatment	8 (4, 14)	8 (3, 13)	12 (8, 18)	< 0.001^3^
Received an Access antibiotic	504 (70%)	446 (69%)	58 (87%)	0.002^2^
Received a Watch antibiotic	429 (60%)	385 (59%)	44 (66%)	0.3^2^
Received a Reserve antibiotic	45 (6%)	36 (6%)	9 (13%)	0.3^4^
Received an antifungal	32 (4%)	24 (4%)	8 (12%)	0.007^4^

^1^Median (IQR) for continuous variables; n (column % of non-missing data) for categorical variables

^2^Pearson’s Chi-squared test

^3^Wilcoxon rank sum test

^4^Fisher’s exact test

Antimicrobial consumption was largely empirical, with 72.4% of DOTs administered in patients without a microbiologically confirmed infection. Prescriptions initiated within 48 h of the start of the follow-up period accounted for 26.8% of all DOTs. Consumption was greatest for co-amoxiclav (168/1000 patient-days), doxycycline (149/1000 patient-days), piperacillin-tazobactam (65/1000 patient-days), and benzylpenicillin (64/1000 patient-days). Of the total 7507 antimicrobial DOTs, 3489 (46.5%) were of Access antibiotics, 3492 (46.5%) of Watch antibiotics, 234 (3.1%) of Reserve antibiotics, according to the UK AWaRe classification [11]; the remaining 292 (3.9%) were of antifungals.

Those with a microbiologically confirmed infection received significantly more days of antimicrobial therapy (17 [10–28] versus 7 [3–12], *p* < 0.001) and greater numbers of antimicrobial courses (5 [3–6] versus 2 [1–3], *p* < 0.001) compared to those without infection. They also accounted for a higher proportion of the DOTs of Reserve antibiotics (47.0%), compared to Watch (34.3%) or Access (17.6%) antibiotics (Fig. 3).

### Association of immunosuppression with infection and antibiotic use

Sixty-seven patients (9%) received high-dose immunosuppression: 50 patients (7%) received high-dose steroids (defined as ≥ 30 mg prednisolone or equivalent for ≥ 2 doses); 13 (2%) received tocilizumab; and 4 (< 1%) received both.

Of the 95 patients who had a co- or secondary infection, 11 (12%) received high-dose immunosuppression, compared to 56 (9%) of the 620 not diagnosed with infection. On multivariable time-dependant analysis, accounting for admission to ICU, age and timing of immunosuppression relative to infection, there was no significant association between receipt of high-dose immunosuppression and subsequent development of a secondary infection (HR 0.56, 95% CI 0.26–1.18).

However, patients receiving high-dose immunosuppression received on average 7.9 more antimicrobial DOTs/1,000 patient-days compared to those who did not (95% CI: 5.0 to 11.0 DOTs). This effect persisted on multivariable analysis: after accounting for ICU admission, co- or secondary infection and duration of follow-up, participants treated with high-dose immunosuppression received 5.5 more DOTs overall (95% CI: 3.4 to 7.7 days). When analysed by antimicrobial category, they received 3.1 more Access DOTs (95% CI: 1.7 to 4.4 days), and 1.0 more antifungal DOTs (95% CI: 0.4 to 1.7 days), with no significant differences in either Watch (95% CI –0.1 to + 2.5 days) or Reserve antibiotics (95% CI –0.2 to + 0.6 days).

## Discussion

Our study at a large London tertiary hospital during the first wave of the UK epidemic found that 84.7% of patients with severe COVID-19 received antimicrobial therapy, despite only 13.3% developing a microbiologically confirmed bacterial or fungal infection. The majority of infections were hospital-acquired, secondary infections with Gram-negative organisms. In those who received immunosuppressive therapy with steroids and/or tocilizumab, we found no evidence of an increased incidence of infection; nevertheless, this group did receive on average 5.5 more days of antimicrobial therapy. Half of all antimicrobial consumption was of agents on the UK AWaRe Antibiotic ‘Watch’ or ‘Reserve’ list, illustrating the need for more judicious antimicrobial prescribing in hospitalised patients with COVID-19.

The low incidence of co-infection in our study (4.2%) is in keeping with the results of a recent meta-analysis (5.3%), although our rates of secondary infection were lower (9.3% vs. 18.4%) [12]. This may be due to our more stringent definition of infection, which required both positive culture and supporting clinical or radiological evidence as determined by an infection specialist. These incidences should also be interpreted in the context of widespread antibiotic prescribing early in the pandemic, which may have protected against secondary infection. Distinguishing secondary infection from progression of COVID-19 pneumonitis is challenging in both a clinical and research setting. Studies that rely purely on culture results likely overstate the incidence of infection, as enteric pathogens commonly colonise the respiratory tract of ventilated patients [13] and their presence does not necessarily indicate infection in the absence of clinical or radiographic features of pneumonia [14]. By interpreting culture results in the context of contemporaneous clinical and radiological findings, we sought to distinguish true infections from culture of commensal organisms.

Gram-negative respiratory and bloodstream infections were most common, with the most frequent pathogens being *Klebsiella spp.*, *Escherichia coli*, and *Pseudomonas aeruginosa*. This is broadly consistent with other studies, although we had notably fewer infections with *Staphylococcus aureus* [4, 12, 15]. In the context of a rapid expansion of bed capacity, there was an outbreak of *Klebsiella pneumoniae* on our ICU during the study period, which may account for the high prevalence of this pathogen [16].

The high rates of antimicrobial prescribing in our cohort (84.7%) are consistent with practice elsewhere during the first pandemic wave [5–7, 17–19]. Doxycycline accounted for the greatest consumption, in line with our local prescribing policy at the time. Despite this, half of all consumption included ‘watch’ and ‘reserve’ antibiotics and prescribing was largely empiric and continued in the absence of confirmed infection. Consumption by antimicrobial class has varied between settings and studies. The UK ISARIC study found that co-amoxiclav was the most frequently prescribed antimicrobial, although consumption metrics were not reported [4]. A large US study found ceftriaxone and azithromycin to be the most commonly used agents [20]. Policies in ten African countries recommended a variety of broad-spectrum antibiotics (azithromycin, ceftriaxone, co-amoxiclav) in initial national COVID-19 guidelines [21]. The upsurge of antimicrobial use in hospitalised patients during the COVID-19 pandemic is likely to have had a detrimental impact on antimicrobial resistance, although quantification of this is challenging [22]. In response to evolving evidence for the low incidence of bacterial co-infection in COVID-19, our local antimicrobial policy now only recommends antibiotics where there is high clinical suspicion of bacterial pneumonia (for example where there is purulent sputum, focal consolidation or neutrophilia prior to steroid administration).

We found a significantly higher consumption of antimicrobials in those who received immunosuppressive therapy, despite finding no increase in the incidence of secondary infection in this group. Concern that immunosuppressive therapies may increase susceptibility to infections might, in part, have driven this prescribing behaviour. The large, pragmatic, multi-platform RECOVERY COVID-19 treatment trial found that immunomodulation with dexamethasone [23] and tocilizumab [24] improved survival in severe COVID-19, transforming subsequent clinical management and outcomes. However, incidence of non-fatal secondary infection was not reported in this trial, nor in another large randomised controlled trial (RCT) of hydrocortisone and tocilizumab [25, 26]. Our study period was prior to the adoption of these therapies as standard of care, allowing a comparison between patients exposed and unexposed to immunosuppressive therapy. We found no evidence of an increased incidence of infection in patients who had received steroids or tocilizumab. Strengths of our study included a stringent and granular approach to the definition of infection and use of time-dependent modelling to account for timing of immunosuppressive therapy relative to infection. Nonetheless, the number of patients exposed to immunomodulatory therapy in our cohort was relatively small and we were unable to account for the impact of antimicrobial use on detection of microbially-proven infection.

Our findings do echo the results of smaller RCTs which collected data on secondary infections and found no increase in incidence in the steroid treatment arms [27–29]. Other retrospective, observational studies, however, have conflicting results, with some suggesting no difference [30] and others suggesting higher infection risk in those receiving steroids [31, 32]. The largest of these studies, which used propensity matching from a cohort of over 4,000 ICU patients, found significantly higher ICU-acquired infections (71% versus 52%, *p* = 0.001) in those who received steroids but not those who received tocilizumab [32]. However, the use of steroids varied significantly between centres, the definition of infection was not standardised and their findings on mortality contradicted those of a major RCT [23]. A WHO meta-analysis of RCTs evaluating IL-6 inhibitors in COVID-19 found no evidence of increased risk of infection (OR 0.99 [95%CI, 0.85–1.16]) [33]. However, another meta-analysis, albeit with less robust inclusion criteria, suggested tocilizumab may increase the risk of fungal infection [34]. Overall, the number of high-quality studies, particularly RCTs, that considered secondary infection as an outcome is small and more robust data are needed.

Our study has several limitations. As a retrospective study, we were reliant on documentation from clinical notes, which may have been incomplete. Our study cohort was single-centre and from the first wave of the pandemic, prior to widespread immunity through vaccination or previous SARS-CoV-2 infection. As use of steroids and tocilizumab was not yet standard-of-care, the indications for these were varied. Of the 17 patients who received tocilizumab, only 4 (24%) received this as part of a randomised trial; the remaining 13 (76%) were part of a compassionate use programme selecting patients with evidence of hyperinflammation [35]. Of those who received high-dose steroids (*n* = 54), only 15 (28%) received these through participation in the RECOVERY trial. Samples were sent for culture according to the clinical team’s standard practice: only 16% of our cohort had respiratory cultures which represents under-sampling.

## Conclusions

The introduction of immunosuppressive therapies has had a dramatic impact on outcomes amongst hospitalised patients with severe COVID-19. Our findings add to the emerging evidence base that co- and secondary infections are uncommon in patients with COVID-19 and that use of immunosuppressive therapies does not appear to confer an increased risk of secondary infection. These findings should galvanise efforts to promote antibiotic stewardship as the COVID-19 pandemic continues.

### Supplementary Information

**Additional file 1: S1.** List of Antimicrobials and Immunosuppressants included in search. **S2.** Automatic Exclusions for non-significant microbiological cultures according to culture site. **S3.** Data Collection Methods. **S4.** Identification of true co- and secondary infections.

## Data Availability

Anonymised, aggregate data analysed during the current study may be available from the corresponding author on reasonable request.

## Abbreviations

CI: Confidence interval
DOT: Days of therapy
HR: Hazard ratio
ICU: Intensive care unit
IL-6: Interleukin 6
OR: Odds ratio
PCR: Polymerase chain reaction
RCT: Randomised controlled trial
WHO: World Health Organisation
