# Supplementary material for: The impact of immunosuppressive therapy on secondary infections and antimicrobial use in COVID-19 inpatients: a retrospective cohort study
Source: BMC Infect Dis. 2023 Nov 17;23:808. doi: 10.1186/s12879-023-08697-9 (PMC10656831; doi:10.1186/s12879-023-08697-9)
Supplement: Supplementary file 1 — Additional file 1: S1. List of Antimicrobials and Immunosuppressants included in search. S2. Automatic Exclusions for non-significant microbiological cultures according to culture site. S3. Data Collection Methods. S4. Identification of true co- and secondary infections. [file 12879_2023_8697_MOESM1_ESM.docx]

**Supplementary Data:**

**S1: List of Antimicrobials and Immunosuppressants included in search**

| **Antimicrobials** | | |
| --- | --- | --- |
| Amikacin  Amoxicillin  Amphotericin  Anidulafungin  Azithromycin  Benzylpenicillin  Caspofungin  Cefalexin  Ceftazidime  Ceftriaxone  Cefuroxime  Ciprofloxacin  Clarithromycin | Clindamycin  Co-amoxiclav  Colistin  Co-trimoxazole  Doxycycline  Ertapenem  Erythromycin  Fidaxomicin  Flucloxacillin  Fluconazole  Fosfomycin  Gentamicin  Levofloxacin | Linezolid  Meropenem  Metronidazole  Moxifloxacin  Nitrofurantoin  Piperacillin  Pivmecillinam  Posaconazole  Teicoplanin  Temocillin  Tigecycline  Trimethoprim  Vancomycin  Voriconazole |
| **Immunosuppressants** | | |
| Dexamethasone  Hydrocortisone  Prednisolone  Methylprednisolone  Tocilizumab  **exposure to immunosuppressant defined as any single dose of tocilizumab or >1 dose of ≥30mg prednisolone or equivalent (≥4.5mg dexamethasone, 120mg hydrocortisone, 24mg methylprednisolone)* | | |

**S2: Automatic Exclusions for non-significant microbiological cultures according to culture site**

| **Respiratory:**   - All *Candida spp.* - Coagulase-negative *staphylococci* - “Upper respiratory tract flora” where not otherwise specified |
| --- |
| **Urine:**   - “Mixed growth” without a predominant species - Coagulase-negative *staphylococci*, except for *Staphylococcus saprophyticus* |
| **Wound swabs:**   - Any isolate other than *Staphylococcus aureus*, *Staphylococcus lugdunensis* or beta-haemolytic *streptococci*, e.g. Group A *streptococcus* |
| *The above results were automatically deemed non-significant without need for further review. All other positive cultures were included for detailed clinical review to assess for true infection.* |

**S3: Data Collection Methods**

| Variables of interest | Data source | Method of assessment |
| --- | --- | --- |
| COVID-related infections | Laboratory information management system (LIMS); electronic patient records (including clinical notes, laboratory data and radiological images) | Medical review |
| Other infection variables (species, site) | As above | As above |
| Antimicrobial Days of Therapy | Electronic prescribing record | Automatic extraction and calculation |
| Other antimicrobial variables (class, number of courses) | As above | As above |
| Receipt of high-dose immunosuppression | Electronic prescribing record | Medical review |
| Demographics (age, gender) and observations (serial oxygen saturations, receipt of supplementary oxygen) | Electronic patient record | Automatic extraction |
| Clinical data (comorbidities, ICU admission, need for organ support, days of hospital stay, days of follow-up, in-hospital mortality) | Electronic patient record | Manual extraction |

**S4: Identification of true co- and secondary infections.**


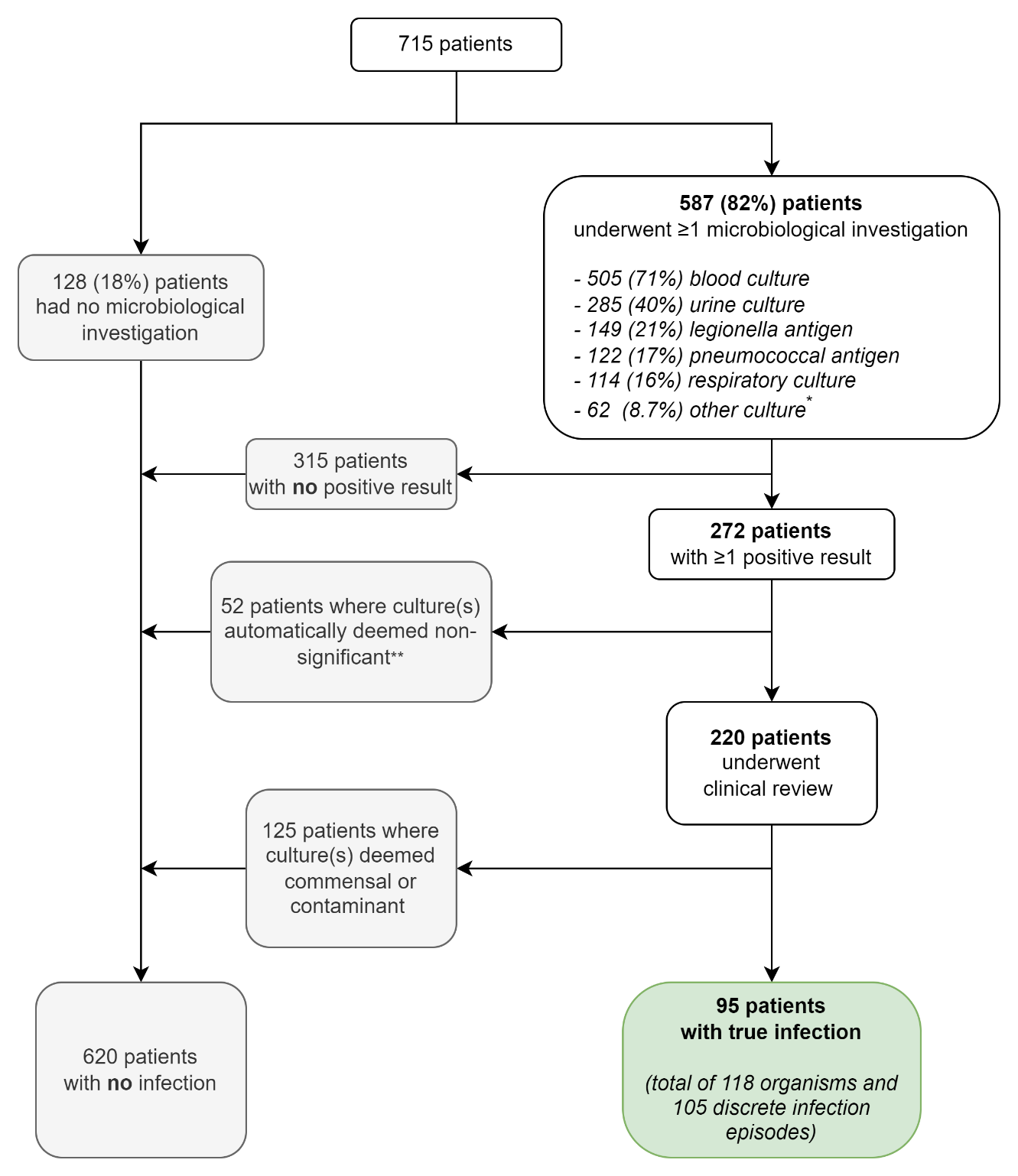


**other cultures (wound swabs, fluid/pus culture, line tip culture) were only recorded where positive so this may be an under-estimate*

*** see Supplementary Table 3 (S3) for list*
